# Supplementary material for: Identification of Variants (rs11571707, rs144848, and rs11571769) in the BRCA2 Gene Associated with Hereditary Breast Cancer in Indigenous Populations of the Brazilian Amazon
Source: Genes (Basel). 2021 Jan 22;12(2):142. doi: 10.3390/genes12020142 (PMC7911168; doi:10.3390/genes12020142)
Supplement: Supplementary file 1 [file genes-12-00142-s001.pdf]

Table S1. Description of all variants in the *BRCA1* and *BRCA2* genes found in the Amerindian individuals (IND).

Gene	SNP ID	Region	Change in nucleotide	Impact predicted by SNPeff	Minor Allele Frequencies					
					IND	AFR	AMR	EAS	EUR	SAS
BRCA1	rs8176318	3UTR	C > A	Modifier	0.2916	0.2263	0.3804	0.4478	0.3655	0.4821
BRCA1	rs799923	Intronic	G > A	Modifier	0.0156	0.0389	0.0845	0.0007	0.2299	0.1797
BRCA1	rs16941	CDS	T > C	Moderate	0.4531	0.1797	0.3083	0.3777	0.3254	0.4996
BRCA1	rs16942	CDS	T > C	Moderate	0.4531	0.2358	0.3141	0.3827	0.3259	0.4999
BRCA1	rs2070833	Intronic	G > T	Modifier	0.0151	-	-	-	-	-
BRCA1	rs1799966	Other[a]	T > C	Moderate	0.4531	0.2397	0.3138	0.3805	0.3268	0.5001
BRCA1	rs12516	3UTR	G > A	Modifier	0.24	0.3012	0.3433	0.4286	0.3841	0.4839
BRCA1	rs1060915	CDS	A > G	Low	0.4531	0.18	0.3088	0.3777	0.3258	0.4993
BRCA1	rs799905	Intronic	G > C	Modifier	0.4754	0.7789	0.4468	0.4403	0.3796	0.5116
BRCA1	rs1799949	CDS	G > A	Low	0.4531	0.2364	0.3138	0.3783	0.3252	0.5002
BRCA1	rs28897689	CDS	T > C	Moderate	0	0.0011	0.0012	-	0.0064	0.0011
BRCA1	rs16940	CDS	A > G	Low	0.4531	0.1792	0.308	0.3785	0.3239	0.4995
BRCA1	rs8176194	Intronic	A > C	Modifier	0	-	-	-	-	-
BRCA1	rs1800062	CDS	C > T	Low	0	0.0002	0.0101	0.0414	0.0001	0.0003
BRCA1	rs779398652	Intronic	CAA > C	Modifier	0	-	-	-	-	-
BRCA1	-	CDS	T > C	Moderate	0	-	-	-	-	-
BRCA1	rs8176235	Intronic	C > T	Modifier	0	-	-	-	-	-
BRCA1	rs1799949	Other[a]	G > A	Moderate	0.4531	0.2364	0.3138	0.3783	0.3252	0.5002
BRCA1	-	Intronic	GTT > G	Modifier	0	-	-	-	-	-
BRCA1	rs799917	CDS	G > A	Moderate	0.4841	0.8193	0.3435	0.378	0.3341	0.5285
BRCA2	rs587780560	Intronic	C > CT	Modifier	0	-	-	-	-	-
BRCA2	rs11571769	CDS	G > A	Moderate	0.1111	0.0012	0.0349	-	0.0044	0.014
BRCA2	rs206076	CDS	G > C	Low	1	0.9253	0.9967	0.9999	0.9995	0.9998
BRCA2	rs1799955	CDS	A > G	Low	0.1065	0.2116	0.1796	0.3847	0.2143	0.2161
BRCA2	rs543304	CDS	T > C	Low	0.0677	0.205	0.1865	0.1792	0.2084	0.1121
BRCA2	rs144848	CDS	A > C	Moderate	0.5078	0.1249	0.3049	0.2728	0.2818	0.3558
BRCA2	rs1799943	5UTR	G > A	Modifier	0.0937	0.1005	0.1895	0.3805	0.2581	0.2829
BRCA2	rs2126042	Intronic	C > T	Modifier	0.1	-	-	-	-	-
BRCA2	rs206075	CDS	A > G	Low	1	0.9264	0.9967	0.9999	0.9995	0.9999
BRCA2	rs1801406	CDS	A > G	Low	0.1031	0.2292	0.2087	0.3848	0.3064	0.2924
BRCA2	rs11571651	Intronic	G > T	Modifier	0	0.0238	0.0633	0.0994	0.0335	0.1157
BRCA2	rs9534262	Intronic	T > C	Modifier	0	0.5729	0.51	0.5544	0.5137	0.4878
BRCA2	rs1801499	CDS	T > C	Low	0.1406	0.0238	0.067	0.1002	0.0348	0.115
BRCA2	rs1799944	CDS	A > G	Moderate	0.1406	0.0427	0.0672	0.1002	0.035	0.1151
BRCA2	rs4987117	CDS	C > T	Moderate	0.1	0.0053	0.0079	0.0001	0.0272	0.0079
BRCA2	rs169547	CDS	T > C	Moderate	1	0.933	0.997	0.9999	0.9996	0.9998
BRCA2	rs11571642	CDS	T > C	Low	0.0833	0.022	0.0022	-	0.0003	-
BRCA2	rs11571707	CDS	T > C	Moderate	0.3203	0.0026	0.1409	0.0036	0.0003	0.0018
BRCA2	rs1801439	CDS	A > G	Low	0.1406	0.0236	0.0667	0.1003	0.0349	0.1156
BRCA2	rs276174816	Intronic	CT > C	Modifier	0	-	-	-	-	-
BRCA2	rs766173	CDS	A > C	Moderate	0.1379	0.024	0.0667	0.1002	0.035	0.1159

[a] NEXT_PROT[modified-residue:phosphoserine]

Table S2: Pairwise FST among Amerindians (IND) and the five continental populations from the 1000 Genomes database.

	IND	AFR	AMR	EAS	EUR	SAS
**IND**	—					
**AFR**	0.17510	—				
**AMR**	0.00301	0.13472	—			
**EAS**	0.03669	0.17347	0.01746	—		
**EUR**	0.04446	0.15776	0.01112	0.02553	—	
**SAS**	0.01700	0.18424	0.03499	0.03956	0.03995	—
